# Motorbike-handlebar hernia - a rare traumatic abdominal wall hernia: a case report and review of the literature

**DOI:** 10.1186/s13256-017-1245-z

**Published:** 2017-03-31

**Authors:** Frank-Leonel Tianyi, Valirie Ndip Agbor, Tsi Njim

**Affiliations:** 1 Sub-Divisional Hospital Mayo Darley, Mayo Darley, Cameroon; 2 Ibal Sub-Divisional Hospital, Oku, North West Region Cameroon; 3 Centre for Tropical Medicine and Global Health, Nuffield Department of Medicine, University of Oxford, Oxfordshire, UK; 4 Health and Human Development Research Group (2HD), Douala, Cameroon

**Keywords:** Handlebar hernia, Traumatic abdominal wall hernia, Case report

## Abstract

**Background:**

Handlebar hernias are very rare and arise following a sudden force from a handle-like object impacting a focal area of the abdomen, which results in a disruption of the underlying abdominal muscle and fascia without necessarily disrupting the overlying skin. Other than a reducible swelling on the abdominal wall, the physical examination of such patients is usually unremarkable and the diagnosis could easily be missed.

**Case presentation:**

An 8-year-old Cameroonian boy with no significant past history presented to our emergency service with a tender left flank swelling following a road traffic accident. He was knocked down by a motorbike with resulting impact of the handlebar on his abdomen. A handlebar hernia was diagnosed on the basis of a reducible abdominal swelling with a positive cough impulse. A herniorrhaphy was done the following day after resuscitation and his postoperative period was uneventful.

**Conclusions:**

Handlebar hernias, although rare, should be suspected when patients present with an abdominal swelling following blunt abdominal trauma involving a handlebar-like object. A good history and physical examination are usually enough to pose an early diagnosis of handlebar hernia. Management typically involves surgical intervention to prevent complications. The timing and surgical approach should be decided on a case-by-case basis.

## Background

Acute abdominal wall hernias caused by traumatic forces are very rare [1, 2]. Handlebar hernias are a more localized type of traumatic abdominal wall hernias, caused by direct trauma from handlebar-like objects [3–7]. They were first described by Landry *et al*. in 1956 [3] and are very rare. A MEDLINE search via PubMed using keywords “handlebar hernia” and “traumatic abdominal wall hernias” revealed 37 case reports on handlebar hernias with none from sub-Saharan Africa. The management involves prompt surgical repair to prevent incarceration or strangulation [8]. We report a case of handlebar hernia in an African boy following a road traffic accident. We elucidate that a good history and physical examination in patients with similar mechanisms of injury could assist in a prompt diagnosis and management in the absence of other diagnostic tools in resource-limited settings.

## Case presentation

An 8-year-old Cameroonian boy with no significant past history was brought to our emergency service following a road traffic accident. On crossing a road, he was knocked down by an oncoming motorbike weighing approximately 125 kg, medium sized, and traveling at moderate speed. The motorbike fell on the boy with the handlebar catching him in the abdomen as its rider lost control. He sustained a lesion to the head with no initial loss of consciousness. On arrival, he complained of pain in his head and a swelling in the left upper quadrant of his abdomen.

**Fig. 1:**
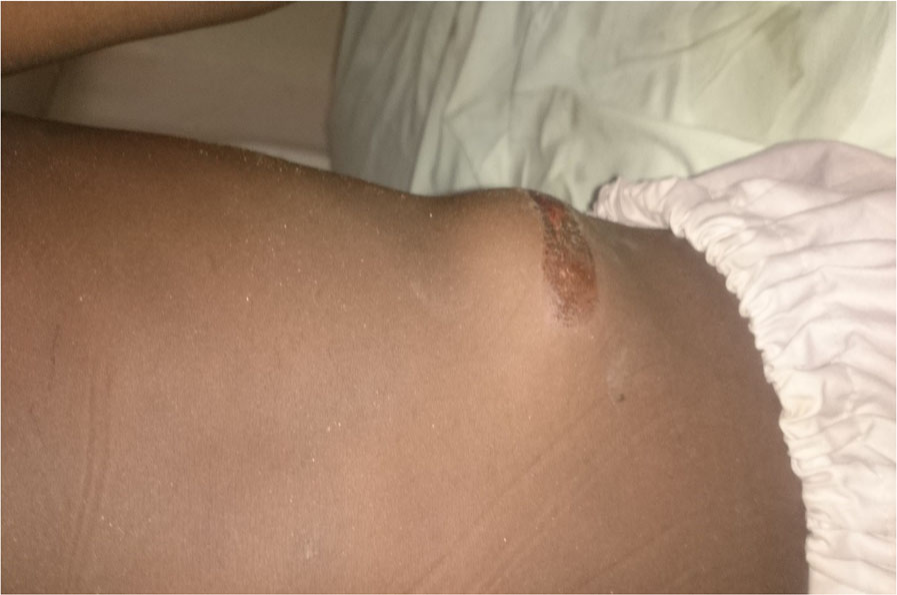
A bulge in the left lumbar region, with bruising of the overlying skin

On examination, he was conscious, with a Glasgow coma score of 15/15, blood pressure of 119/83 mmHg, pulse of 90 beats per minute, respiratory rate of 18 breaths per minute, temperature of 37.3 °C; he was 96 cm tall and weighed 26 kg. There was a 2 cm laceration in the occipital region of his head, and a swelling in his left lumbar region with mild bruising of the overlying skin (Fig. 1). The swelling was reducible and disappeared in the supine position. There was no guarding and no rebound tenderness. He had normal bowel sounds on auscultation and the rest of the examination was unremarkable.

**Fig. 2:**
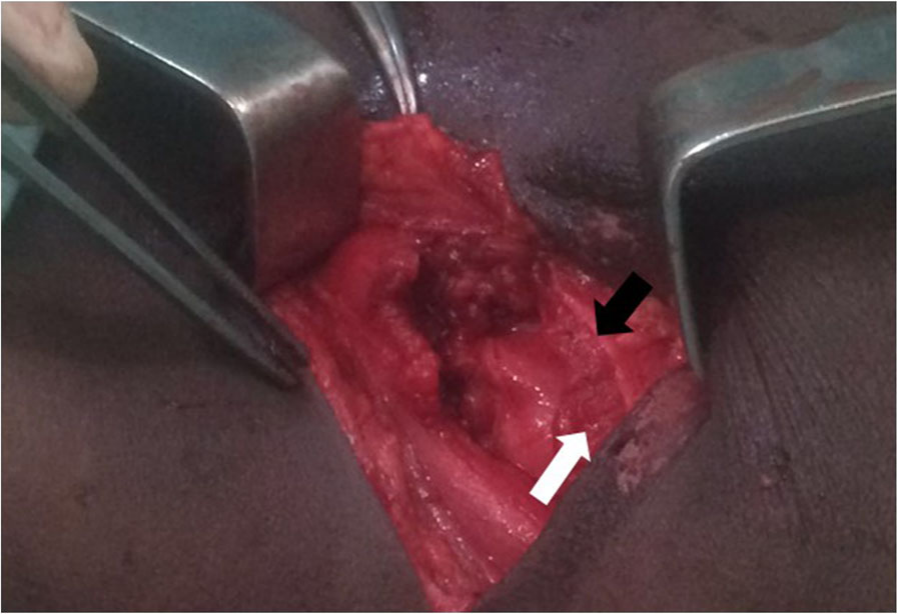
Disruption of the layers of the lateral abdominal wall showing the fascia (*black arrow*) and muscles (*white arrow*)

A diagnosis of mild head injury and a handlebar hernia was made. After resuscitation, his scalp laceration was closed with a one point suture and he was programmed for surgery for the handlebar hernia the next day. A surgical exploration of the area showed a 7 cm laceration of his lateral abdominal wall (Fig. 2), running from the external oblique aponeurosis through all the muscle layers to his peritoneum with loops of small bowel present in the opening. There was no blood or feces in the area immediately surrounding the lesion. Exploration of the bowel loops closest to the opening did not reveal signs of intra-abdominal injury. The abdominal wall defect was repaired in layers. The abdominal incision was closed in a standard manner. His postoperative period was uneventful. He was discharged on postoperative day 9 and scheduled 2 weeks later for a follow-up visit, but unfortunately was lost to follow-up.

## Discussion

A traumatic abdominal wall hernia is defined as a herniation through disrupted musculature and fascia, following considerable trauma, without skin penetration and no evidence of a prior hernia defect at the site of injury [9]. They arise from blunt forces directed normally to the abdominal wall, with a resulting pressure-induced disruption of the underlying muscles, fascia, and peritoneum [10, 11]. Being more elastic, the skin remains intact, even though the muscle and fascia are disrupted, leading to a traumatic abdominal wall hernia [7, 12]. Wood *et al*. [13] classified traumatic abdominal wall hernias into three types, depending on the size of the rupture and cause of the injury. Type I abdominal wall hernias involve a small defect caused by blunt trauma. Type II hernias are larger defects developed during high-energy transfer events such as motor vehicle crashes or falls from heights. Type III hernias are those defects that involve intra-abdominal bowel herniation and have been described for deceleration injuries [13–15]. Handlebar hernias fall under type I abdominal wall hernias and are rarely associated with intra-abdominal injuries [3, 14, 16]. Following trauma, a localized blunt force is transmitted through a small focal point, a handlebar in this case, to the abdomen. Being blunt, the force transmits energy through the skin without penetrating it and the transferred energy dissipates through the abdominal wall, which results in a splitting of the muscle fibers without penetration [17, 18]. A low energy injury like ours will cause a hernia when the force is directed at regions of anatomic weakness such as the inguinal region or the lower abdomen lateral to the rectus muscle [17]. Consequently, type I traumatic abdominal wall hernias rarely occur in the midline because the tough fascial envelope affords some protection from this type of injury. The abdominal wall of a child has a thin musculature and this places them at a higher risk of developing a type I hernia following contact between the abdominal wall and handlebar-like objects [19]. Our patient sustained the injury following contact between his abdomen and the handlebar of the motorbike as it fell to the ground. This caused a defect in his lateral abdominal wall with an intact overlying skin. The mechanism and the injury are coherent with a type I abdominal wall hernia, hence our diagnosis of a handlebar hernia.

Of the 37 case reports on handlebar hernia retrieved through a PubMed search, 29 (78%) occurred in children aged 4 to 14 years [5, 7, 9, 15, 20]. In adults, the hernias were observed to occur at anatomical weak points such as in the inguinal region or the lower abdomen lateral to the rectus [14, 17]. The location in children was more varied, occurring mostly in the lower abdomen, with two reports in the upper abdomen [5, 21] and one over the rectus abdominis muscle [18]. This highlights the thin and weaker abdominal wall of children as a cause of the higher frequency of this injury among this population.

The diagnosis of handlebar hernia is mostly clinical [14, 18]. A history of blunt trauma to the abdomen should alert the physician to the possibility of an abdominal wall hernia. On examination of the abdomen, there may be bruising around the affected area with an imprint sometimes visible in fair skinned individuals. Around the bruising, there is a tender swelling, which is often reducible, with a cough impulse. Other than the above findings, the rest of the abdominal examination is usually normal. In severe cases involving damage to the intestines, there could be signs of peritonitis such as abdominal wall guarding, rigidity, and rebound tenderness [18]. In some rare instances, it is possible to have a hernia without any of the above signs. There are cases of patients in which symptoms presented 48 hours after the accident [22]. In these situations, additional imaging in the form of ultrasounds and computed tomography scans can be of help, given that they facilitate the diagnosis, define the anatomy of the disrupted abdominal wall layers, evaluate intra-abdominal injuries, and differentiate a hernia from a hematoma which is a common differential diagnosis [1, 14, 18]. In our case, our diagnosis was based solely on history and a physical examination. This supports the notion that a high index of suspicion in clinicians following a thorough history and physical examination could lead to the prompt diagnosis of the condition in the absence of other imaging techniques in settings with limited resources. Early diagnosis could guide appropriate management and prevent further morbidity and/or mortality.

The management of handlebar hernias involves prompt surgical repair to prevent incarceration or strangulation [8, 23]. If the defect is small and localized, repair can be achieved with primary closure of torn layers with non-absorbable sutures. With larger defects prosthetic materials are often used for repair [18]. Controversies surrounding the surgical management of traumatic abdominal wall hernias are centered on two main points: the timing of the surgery and the best surgical approach [14, 18].

The timing of the surgery could be emergent or delayed. The most important factor for the timing of the surgery is the probability of intra-abdominal injury, with immediate operation being preferred as it allows the ruling out of any intra-abdominal injury and prevents strangulation of any incarcerated bowel [20]. However, if there is no immediate risk of strangulation and there are no signs of intra-abdominal injury, it might be corrected on a delayed basis [8], with there being a case of successful conservative management [24].

It is important to always rule out intra-abdominal injury when managing traumatic abdominal wall hernias. There exists a debate on whether a midline exploratory laparotomy or local wound exploration is best to rule out intra-abdominal injury [15]. In the presence of signs of intra-abdominal injury, a midline exploratory laparotomy is necessary to facilitate exploration and manage all associated injuries [14]. In the absence of signs of intra-abdominal injury such as blood or feces in the peritoneal cavity, a local wound exploration and primary wound closure usually suffice [18]. Simplified numerical models [25] and finite element models [26] are currently being developed in order to be able to establish the probability of injury on the basis of impact speed and location. Our patient was managed by local wound exploration, after we did not find any signs suggestive of intra-abdominal injury. We, however, recommend a selection of the best approach on a case-by-case basis.

## Conclusions

Handlebar hernias, although rare, should be suspected when patients present with an abdominal swelling following blunt abdominal trauma involving a handlebar-like object. In the absence of an ultrasound and computed tomography scan, a good history with a clear mechanism of injury and a physical examination are imperative for an early diagnosis. Management typically involves surgical intervention to prevent complications, but the timing and the surgical approach should be decided on a case-by-case basis.
